# Early Structure–Function Correlates in Neovascular Age-Related Macular Degeneration Using Artificial Intelligence–Derived OCT Biomarkers

**DOI:** 10.1016/j.xops.2026.101281

**Published:** 2026-06-10

**Authors:** Virginia Mares, Stefan Sacu, Sophie Frank-Publig, Anna Eidenberger, Philipp Fuchs, Leonard M. Coulibaly, Magdalena Baratsits, Georg Faustmann, Markus Gumpinger, Emese Sükei, Hrvoje Bogunovic, Gregor S. Reiter, Oliver Leingang, Alexandra Miere, Catherine Creuzot-Garcher, Laurent Kodjikian, Vincent Gualino, Benjamin Wolff, Ulrike Scheschy, Ursula Schmidt-Erfurth

**Affiliations:** 1 Department of Ophthalmology, Medical University of Vienna, Vienna, Austria; 2 Department of Ophthalmology, Federal University of Minas Gerais, Belo Horizonte, Brazil; 3 Department of Ophthalmology, Christian Doppler Lab for Artificial Intelligence in Retina, Medical University of Vienna, Austria; 4 RetInSight, Vienna, Austria; 5 Department of Ophthalmology, Intercity Hospital of Créteil, University Paris-Est Créteil, France; 6 Department of Ophthalmology, Dijon University Hospital, Dijon, France; 7 Department of Ophthalmology, Croix Rousse University Hospital, Hospices Civils de Lyon, Lyon, France; 8 Department of Ophthalmology, Clinique Honoré-Cave, Montauban, France; 9 Maison Rouge Eye Center, Strasbourg, France; 10 Department of Ophthalmology, Horn Regional Hospital, Horn, Austria

**Keywords:** Biomarkers, Deep learning, Neovascular age-related macular degeneration, OCT, Visual acuity

## Abstract

**Purpose:**

To correlate automated artificial intelligence–based retinal fluid quantification with ellipsoid zone (EZ) thickness and loss, macular neovascularization (MNV) type, and visual function.

**Design:**

This is a post hoc analysis of a prospective, real-world, multicenter clinical trial.

**Subjects:**

Patients with active neovascular age-related macular degeneration (nAMD) were included.

**Methods:**

Best-corrected visual acuity (BCVA), measured using the ETDRS chart, OCT, OCT angiography, and microperimetry, were collected by the same certified examiners. Subretinal fluid (SRF), intraretinal fluid (IRF), and pigment epithelial detachment (PED) volumes for the central 1- and 6- mm areas were calculated at baseline using the Fluid Monitor (Retinsight) on Spectralis OCT (Heidelberg Engineering). An automated algorithm segmented EZ thickness and loss detectable after fluid resolution at month 1.

**Main Outcome Measures:**

Fluid volumes were associated with MNV type, EZ thickness and loss, retinal sensitivity, and BCVA using univariate and multivariate regression models and Wilcoxon rank-sum tests with bootstrapped confidence intervals. Spearman correlation was used to analyze retinal sensitivity and EZ loss.

**Results:**

Two hundred ninety eyes from 290 consecutive patients with active MNV were included in the study at baseline. Macular neovascularization type 1 was the most prevalent in this cohort. Comparing MNV types, lower amounts of IRF were found in type 1 (all *P* < 0.001) and lower amounts of SRF for type 3 (all *P* < 0.05), in the central 1- and 6-mm areas. A decreased BCVA at month 1 was associated with IRF in the central 1 mm at baseline (–6.8 letters/100 nL; *P* < 0.001). Intraretinal fluid and PED volumes at baseline were correlated with EZ loss at month 1 in the central 6 mm. High fluid volumes of IRF, SRF, and PED in the central 1- and 6- mm areas also correlated with decreased mean retinal sensitivity (all *P* < 0.01). Ellipsoid zone loss in the central 1- and 6-mm area correlated with decreased retinal sensitivity (all *P* < 0.01).

**Conclusion:**

Retinal fluid volumes in nAMD are triggered by MNV types and correlate with photoreceptor integrity and visual function, with the impact depending on the fluid compartment. Although fluid is central to disease monitoring, a more comprehensive approach to structural-functional assessment in nAMD is of interest.

**Financial Disclosure(s):**

Proprietary or commercial disclosure may be found in the Footnotes and Disclosures at the end of this article.

Neovascular age-related macular degeneration (nAMD) is responsible for nearly 90% of the severe vision loss in patients with age-related macular degeneration.^1^ Retinal fluid volumes correspond to the most critical biomarker in the management of nAMD, and its presence is still a key factor driving treatment decisions in clinical practice.^2^ Despite regular treatment, end-stage anatomic sequelae such as subretinal fibrosis or macular atrophy (MA) may occur, demonstrating the role of individual components in disease progression and retinal fluid behavior.^3^ Results from the Comparison of AMD Treatment Trials study showed that vision gains during the first 2 years were not maintained at the 5-year follow-up, regardless of the treatment regimen adopted.^4^ A recent meta-analysis reported a cumulative incidence of 31% of fibrosis,^5^ and an incidence rate of 29% of macular atrophy at the 2-year follow-up.^6^ The Inhibition of VEGF in Age-related choroidal Neovascularisation trial showed that MA was observed in almost 90% of eyes after 6 years of follow-up.^7^ Although the development of MA in nAMD is not yet fully understood, it has been associated with several factors, including the presence of intraretinal fluid (IRF), reticular pseudodrusen, and the collapse of pigment epithelial detachment (PED).^8^, ^9^, ^10^ Therefore, early detection of atrophy signs such as loss of the ellipsoid zone (EZ) can assist in more efficient management of the patients and improve disease understanding.

OCT is the gold standard imaging method to monitor nAMD. However, the interpretation of OCT images is subjective, and quantitative changes in fluid dynamics or other biomarkers such as retinal layers cannot be accurately evaluated by clinicians. To address these challenges, the integration of artificial intelligence (AI)–based fluid monitoring algorithms for nAMD has emerged as a promising solution.^11^^,^^12^ Most importantly, AI can not only quantify fluid but also the photoreceptor condition, that is, EZ thinning and loss.^13^, ^14^, ^15^, ^16^ The ability to precisely quantify and localize retinal fluid volumes demonstrated that fluid type, fluid volumes, and their fluctuation play an important role in nAMD progression, with IRF being more often associated with worse visual and anatomic outcomes.^14^^,^^17^^,^^18^ Despite its clinical relevance, fluid alone does not fully explain the variability in functional outcomes. Several patients with minimal or resolved fluid continue to exhibit poor visual function, whereas others with persistent fluid maintain relatively good vision.^19^ These findings highlight that retinal fluid is 1 component of a multifactorial degenerative process, and early structure–function correlation should assess not only the fluid but also external retinal bands such as EZ integrity.

This study performed a robust early structure–function correlation, including fluid volume distribution per compartment, together with EZ thinning and loss and correlation with best-corrected visual acuity (BCVA) and retinal sensitivity using validated AI-based fluid and EZ monitoring. The study was performed, for the first time, in a large population, which is representative of a clinical setting; therefore, the results elucidate the real potential impact of AI-based structure–function correlation on clinical practice, patient outcomes, and healthcare resource utilization.

## Methods

### Participants

Patients with active nAMD were included in a prospective, multicenter, randomized (1:1), double-masked phase III clinical trial registered in the European Union Drug Regulating Authorities Clinical Trials Database (number: 2019-003133-42).^20^ In this study, data from baseline and month 1 were used for randomization, and treatment assignment was performed only during the subsequent visits. The study has been approved by the Ethics committee of the Medical University of Vienna (EK Nr: 1672/2019) adhering to the Declaration of Helsinki and all participants have given written informed consent prior to inclusion. Informed consent was signed by each patient. Best-corrected visual acuity worse than 20/200 on the Snellen chart equivalent, inactive nAMD, and presence of macular atrophy or subretinal fibrosis in the central 1-mm area at baseline were not included. Eyes with concomitant macular diseases such as macular holes and diabetic macular edema, late-stage glaucoma (with cup-to-disc ratios > 0.9), presence of corneal decompensation, haze or scarring with an impact on BCVA were excluded. Furthermore, patients with a history of surgical procedures on the study eye within the last 3 months before baseline or any time after inclusion were excluded. Monthly study visits were performed in which demographic and clinical data were collected, as well as BCVA, color fundus photography, spectral-domain (SD) OCT (Spectralis, Heidelberg Engineering), and OCT angiography (OCTA) (PLEX Elite 9000, Carl Zeiss Meditec, Inc). Microperimetry (MP) was performed using MP-3 microperimeter (Nidek Co). Anonymized SD-OCT images were segmented using the Fluid Monitor (RetInsight).^21^, ^22^, ^23^

To evaluate the impact of retinal fluid at baseline on BCVA and EZ integrity after the first injection, the patients were divided into 2 groups. The highest 25% quartile of fluid volume in each compartment IRF and subretinal fluid (SRF) and PED at baseline in the central 6 mm were classified as the high fluid volume subgroup. The remaining 75% of patients were classified as the low fluid volume subgroup for each respective compartment.^21^^,^^24^

### Visual Acuity, Macular Neovascularization Type, Macular Sensitivity and Automated Quantification of Retinal Fluid, and EZ Loss

Best-corrected visual acuity was tested at every visit using the ETDRS chart by the same certified examiners. OCT imaging acquisition was performed using Spectralis 20° × 20°, 97 sections, high-resolution, automatic real-time 16 frames. OCT angiography slab-based en face assessment with manual verification of segmentation boundaries for macular neovascularization (MNV) subtype classification was performed. Macular neovascularization type classification was determined based on OCT and OCTA by the certified attending physician at each center. Type 1 MNV was identified by neovascular growth posterior to the retinal pigment epithelium (RPE), often associated with PED. On OCTA, these lesions appeared as a vascular network beneath the RPE. The type 2 MNV features neovascularization that grows above the RPE into the subretinal space, appearing as a hyperreflective lesion on OCT, with OCTA showing a distinct vascular network above the RPE. Type 3 MNV, also known as retinal angiomatous proliferation, is characterized by intraretinal neovascular growth that may extend toward the outer retina, with OCT displaying intraretinal cysts, hemorrhage, or hyperreflective spots. OCT angiography visualizes a vascular proliferation originating from the deep retinal layers toward or even penetrating the level of the RPE.^25^ Baseline characteristics and MNV subtype prevalence are described in Table 1. Macular sensitivity was assessed at baseline using the MP-3 microperimeter over 45 central test points covering the ETDRS grid.

**Table 1:** Baseline Characteristics of 290 Patients Included in the Study

Variable		Frequency	
		N	%
Gender	Male	177	61
	Female	113	39
Age	Mean ± standard deviation	79.09 ± 7.34	
Treatment naïve	Yes	134	46.2
	No	156	53.8
BCVA at baseline	Mean ± standard deviation	74.5 ± 9.9	
MNV subtype	Type 1	194	66.9
	Type 2	29	10
	Type 3	67	23.1

BCVA = best-corrected visual acuity; MNV = macular neovascularization.

**Figure 1:**
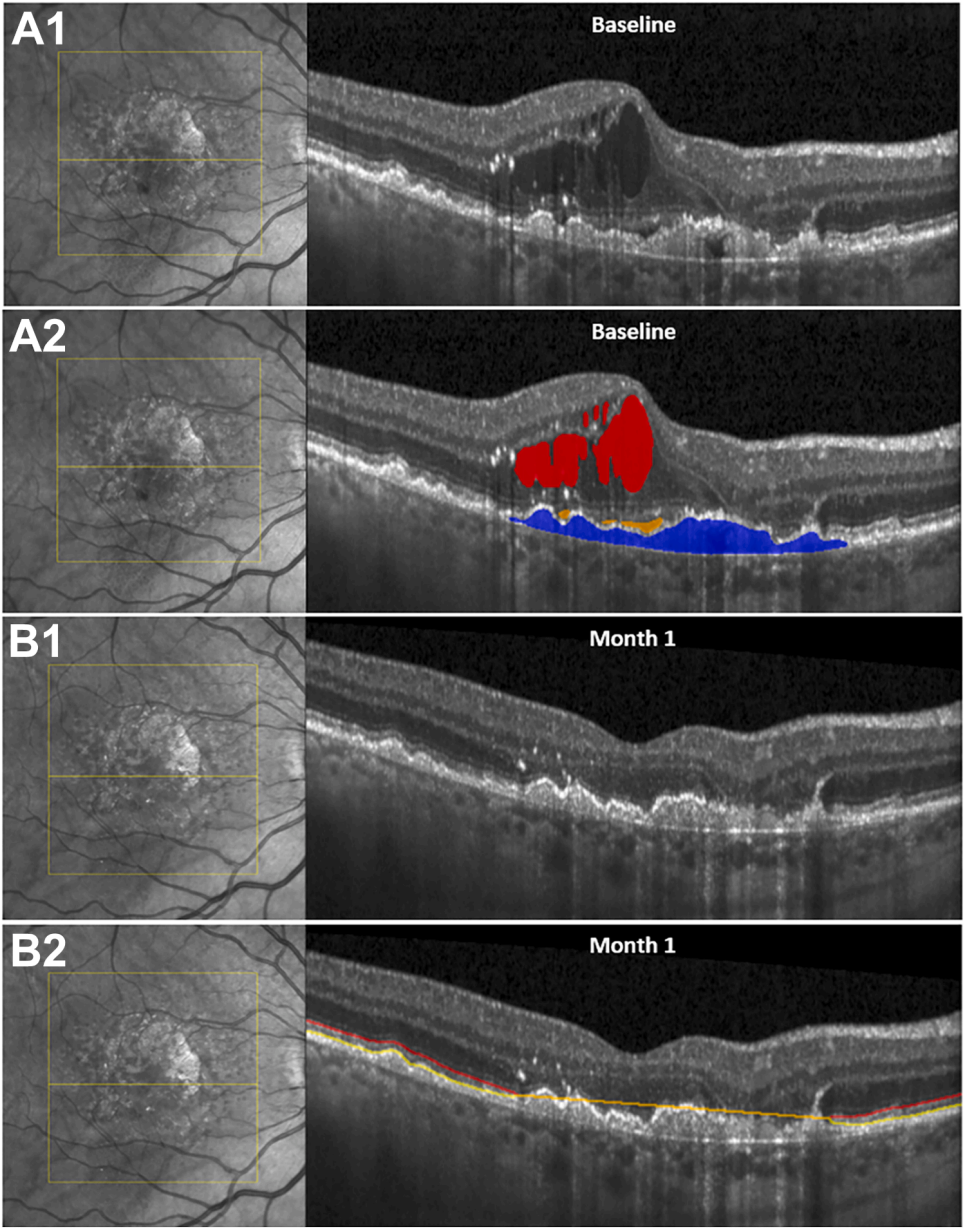
Fluid segmentation at baseline and ellipsoid zone segmentation at month 1. Intraretinal fluid (red), subretinal fluid (yellow), and pigment epithelial detachment (blue) are shown in Figure A2. Ellipsoid zone thickness is segmented as the inner border of the ellipsoid zone (red line) and the outer boundary of the interdigitation zone (yellow line). In areas where ellipsoid zone loss occurs, the lines overlap and appear as a single dark yellow line as shown in Figure B2.

Intraretinal fluid, SRF, and PED volumes within the central 1- and 6-mm macular subfields were automatically segmented and quantified using a certified deep learning algorithm (Fluid Monitor, RetInSight) at baseline and at month 1 (Fig 1). The algorithm uses a convolutional neural network to identify retinal fluid and PED on a pixel-level and has been validated in nAMD, including large data sets.^26^ The number of assigned pixels can be computed into an estimate of fluid volumes in nanoliters (1 nL = 0.001 mm^3^) in their respective compartments.^12^^,^^27^ Ellipsoid zone integrity was assessed at month 1 instead of baseline, when retina fluid volumes are reduced or even absent, to ensure that no overlying fluid-related masking or reflection would compromise an accurate EZ quantification. The EZ layer was defined as continued segmentation of the area between the top of the EZ and the outer boundary of the interdigitation zone. Automated segmentation of EZ thickness was performed by a previously reported ensemble of U-Net–based fully convolutional neural network delineating layers on each individual B-scan of the entire SD-OCT volume.^28^^,^^29^ Ellipsoid zone loss was computed as area (mm^2^) within the respective regions in which EZ thickness is not detectable. The EZ segmentation algorithm has been previously validated by Orlando et al^28^^,^^30^ in a mixed cohort including age-related macular degeneration and tested in a clinical setting nAMD cohort from the FRB data set with accurate performance.^14^ The native output of the EZ algorithm is EZ thickness, and EZ loss is identified when EZ thickness is no longer identifiable (Fig 1). All SD-OCT volumes were subsequently reviewed for image and segmentation quality, and 60 were manually corrected by 2 experienced senior readers. Baseline retinal sensitivity was also correlated with fluid volumes at baseline and with EZ integrity at month 1.

### Statistical Analysis

Associations of baseline macular fluid volumes with MNV type, EZ thickness, and EZ loss at month 1, BCVA measured in ETDRS letters, and retinal sensitivity were assessed using univariate and multivariable linear regression models. Fluid volumes were analyzed both as continuous predictors and after stratification into high (top 25%) and low (bottom 75%) subgroups for each compartment (IRF, SRF, and PED) and region (1 and 6 mm) at baseline to capture clinically high fluid burdens while retaining sufficient power in the comparator group. The distributions of fluid volumes were non-Gaussian; therefore, comparisons of high- versus low-fluid subgroups were performed using Wilcoxon rank-sum tests, with Bonferroni correction applied to control the family-wise error rate at 0.05 for multiple comparisons. Variance inflation factors were calculated for IRF, SRF, and PED volumes (analyzed separately for the 1- and 6-mm regions), with variance inflation factor close to 1, demonstrating little to no multicollinearity. Multivariable regression models were then fitted, including fluid volumes together with age, sex, baseline BCVA, treatment-naïve status, and MNV subtype to evaluate independent associations with BCVA, EZ thickness and loss, and retinal sensitivity. For BCVA and EZ outcomes, additional mixed-effects models with a random intercept for study center were used to account for site-level clustering. Interreader agreement was assessed at a B-scan level using intraclass correlation coefficients (ICCs) based on a 2-way random-effects model with absolute agreement. Intraclass correlation coefficients were calculated for single measurements (ICC[2,1]) and for the mean of readers (ICC[2,k]).

## Results

Two hundred ninety eyes from 290 patients were included in the study at baseline, of whom 177 (61%) were female, and 134 (46.2%) were treatment-naive. The mean age was 79.09 (standard deviation, ±7.34), and the mean BCVA at baseline was 74.4 (standard deviation, ±9.99) letters (Table 1).

**Figure 2:**
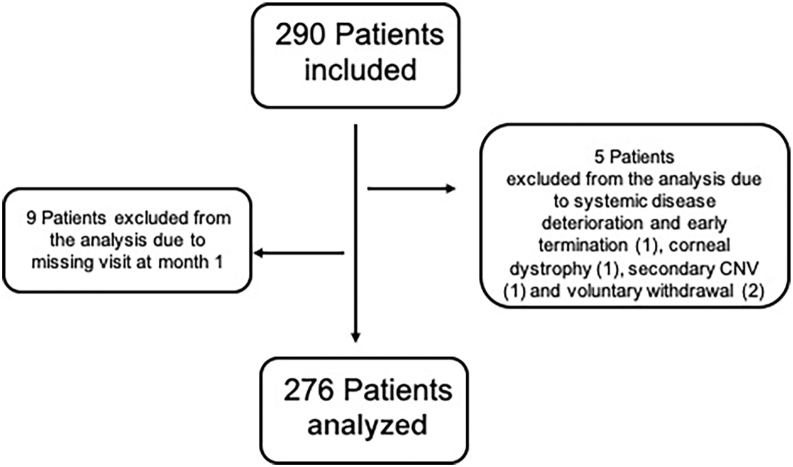
Diagram illustrating the study cohort selection and exclusion criteria.

The mean IRF, SRF, and PED volumes at baseline were 15, 19, and 62 nL in the central 1 mm, and 55, 190, and 607 nL in the central 6 mm, respectively. Macular neovascularization type I was present in 194 eyes (67%), type 2 in 29 eyes (10%), and type 3 in 67 eyes (23%) of the patients. To correlate fluid volumes at baseline with BCVA and EZ loss at month 1, 276 patients imaged at month 1 were included in the study. Of the 290 patients included in the study, 5 patients were excluded from the analysis, and 9 patients missed the visit at month 1 (Fig 2).

**Figure 3:**
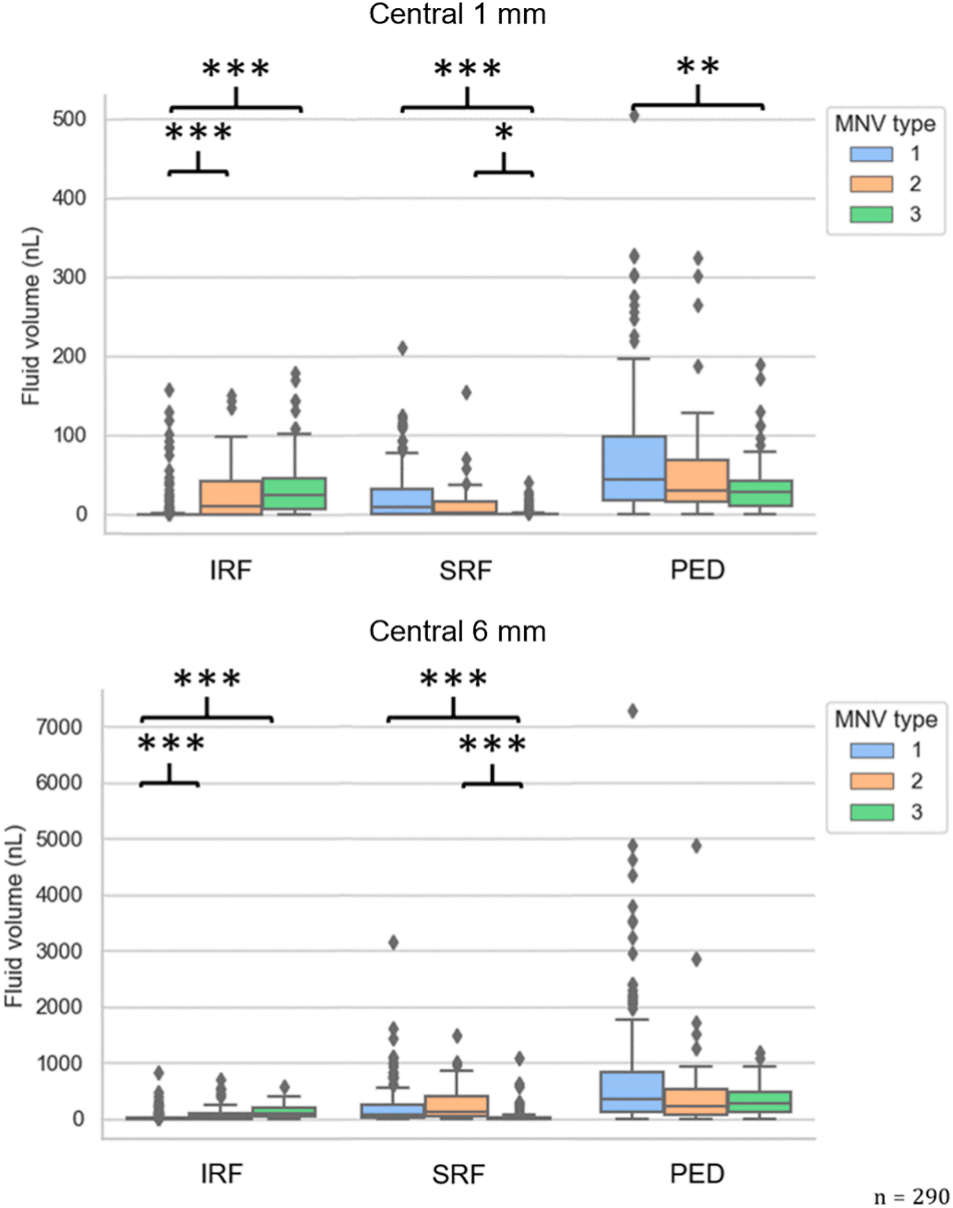
Distribution of intraretinal fluid (IRF), subretinal fluid (SRF), and pigment epithelial detachment (PED) volumes in the central 1-mm (top) and 6-mm (bottom) regions across different macular neovascularization (MNV) types. ∗*P* < 0.05; ∗∗*P* < 0.01; ∗∗∗*P* < 0.001.

Comparing MNV types and prevalence of fluid volumes in each compartment (Fig 3), in both the central 1- and 6-mm regions, IRF volumes were significantly lower in type 1 (all *P* < 0.001), and SRF was significantly lower in type 3 (all *P* < 0.05).

A multivariable linear regression analysis with covariate adjustment of age and sex showed that IRF in the central 1- and 6-mm regions at baseline was associated with a decrease in visual acuity of –6.8 and –2.1 letters/100 nL (all *P* < 0.001), respectively. When adding baseline BCVA, treatment-naïve status, and MNV type to the model, only SRF in the central 6-mm region was subtly associated with an increase of 0.3 letters/100 nL at month 1 (*P* = 0.007). Treatment naïve alone did not impact the initial results. Pigment epithelial detachment volumes showed no statistically significant impact on BCVA in month 1. Variance inflation factors were 1.0 for IRF, SRF, and PED, indicating no multicollinearity. Analyzing high- versus low-fluid volume subgroups per compartment, there was no independent association between higher IRF, SRF, and PED and BCVA outcome after the first injection.

Total retinal fluid volumes at baseline presented an impact on EZ thickness and EZ loss at month 1. A higher total fluid volume in the central 6-mm area at baseline was significantly related to EZ loss at month 1 (*P* < 0.001). The analysis per compartment (Fig 3) showed that IRF and PED volumes were significantly associated with EZ loss in the central 1 and 6 mm. The highest impact was related to IRF with 0.09 mm^2^ of EZ loss per 10 nL (*P* < 0.001) in the central 6 mm. The multivariate regression analysis with covariate adjustment of age, sex, baseline BCVA, treatment naïve status, and MNV type presented similar results. Subretinal fluid showed no statistically significant association with EZ loss. Regarding EZ thickness, SRF presented a slight increase of 0.03 micron per 10 nL (*P* < 0.001) in the central 6 mm. Given the potential bias in EZ segmentation in the presence of SRF, we repeated the analysis after excluding eyes with macular SRF at month 1. A total of 122 eyes were included. In multivariable regression models adjusted for all previously specified confounders, SRF remained associated with increased EZ thickness (β = 0.04; 95% confidence interval [CI], 0.01–0.07; *P* = 0.02).

**Figure 4:**
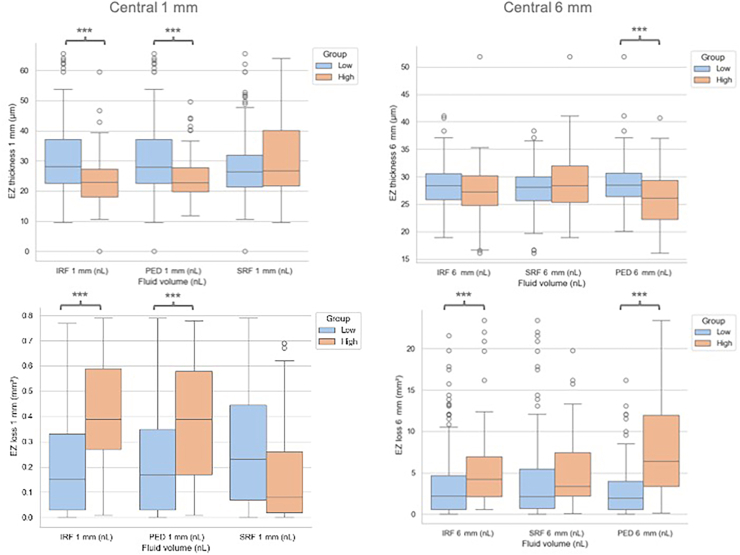
Comparison of ellipsoid zone (EZ) thickness and EZ loss between low- and high-volume subgroups of intraretinal fluid (IRF), subretinal fluid (SRF), and pigment epithelial detachment (PED) in the central 1 and 6 mm of the macula. ∗*P* < 0.05; ∗∗*P* < 0.01; ∗∗∗*P* < 0.001.

**Figure 5:**
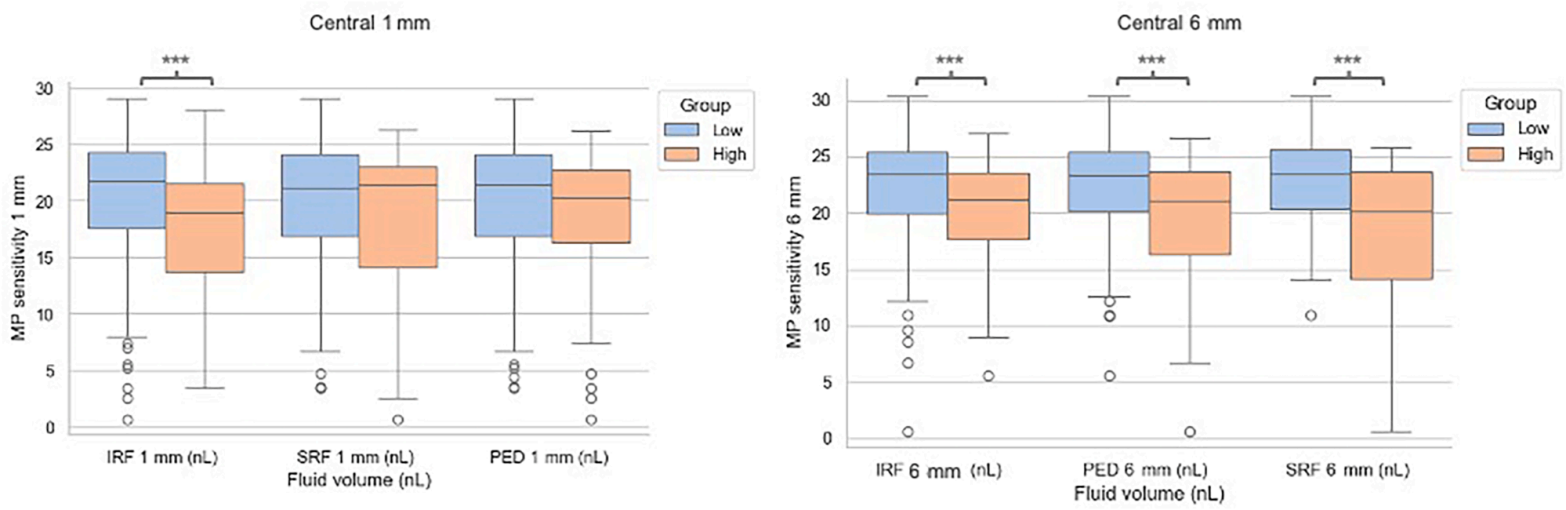
Comparison of mean retinal sensitivity between fluid volume subgroups per compartment in the central 1 and 6 mm of the macula. Microperimetry (MP), subretinal fluid (SRF), intraretinal fluid (IRF), and pigment epithelial detachment (PED). For mean sensitivity of the central 1 mm disc, the mean of the central 9 stimulation points from the MP examination (MP3) was calculated.

**Figure 6:**
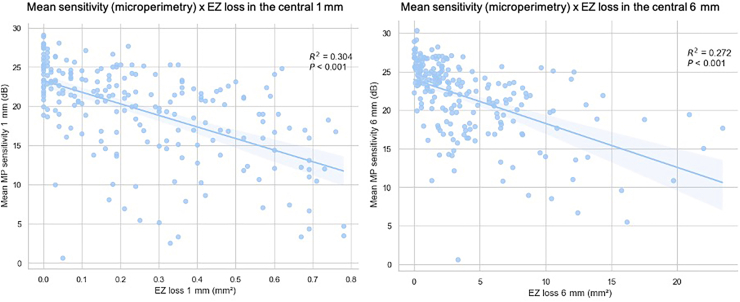
Scatter plot showing the negative association of ellipsoid zone (EZ) loss and mean sensitivity. MP = microperimetry.

The regression analysis revealed a positive correlation between EZ thickness and BCVA and a negative correlation between EZ loss and BCVA in the central 1 mm (*R*^2^ = 0.175) and 6 mm of the macula (*R*^2^ = 0.08), all *P* < 0.001. The higher explained variance, represented by the *R*^2^ values observed in the central 1-mm compared with the 6-mm area, likely reflects the foveal higher density of photoreceptors, therefore a stronger anatomical–functional correspondence. Furthermore, larger macular areas may also increase lesion heterogeneity. High fluid volumes of IRF, SRF, and PED were significantly associated with reduced mean retinal sensitivity within the central 1- and 6-mm areas (all *P* < 0.01). Among these, IRF in the central 1 mm demonstrated the strongest association, with a reduction of –5.6 dB in retinal sensitivity per 100 nL increase in volume (Table 2). Figure 4 illustrates the comparison between higher and lower fluid volume subgroups. Among the higher and lower fluid volume subgroups, retinal sensitivity was significantly lower in eyes with higher volumes of IRF, SRF, and PED within the central 6 mm. Within the central 1 mm, only IRF volume remained associated with reduced sensitivity between subgroups. Baseline retinal sensitivity was associated with EZ loss measured at month 1 in the central 1 and 6 mm (all *P* < 0.001) of the macula. This association remained positive, albeit modest, in a multiple linear regression model including both EZ loss and total fluid volume as independent variables in the central 1 and 6 mm (*R*^2^ = 0.262 = 0.392, respectively, all P < 0.001) (Fig 5). Beyond mean sensitivity, the analysis of MP loci with deep visual sensitivity defects, specifically ≤10 dB, is consistently associated with structural damage. The proportion of MP loci ≤10 dB showed positive associations with EZ loss in both the central 1- and 6-mm regions (0.03 and 0.28 mm,^2^ respectively, all *P* < 0.001) (Fig 6).

**Table 2:** Association between Retinal Fluid Volume per Compartment and Visual Acuity, Ellipsoid Zone Loss and Mean Retinal Sensitivity in the Central 1 and 6 mm of the Macula

Fluid Compartment	Model	Region	BCVA (Letters/100 nL) 276 Eyes	EZ Loss (mm^2^/10 nL) 276 Eyes	Retinal Sensitivity (dB/100 nL) 224 Eyes
IRF	Adjusted for age and sex	1 mm	–6.8 (*P* < 0.001) 95% CI (–10.1 to –3.4)	0.03 (*P* < 0.001) 95% CI (0.02–0.03)	–5.6 (*P* < 0.001) 95% CI (–7.6 to –3.6)
	Adjusted for all covariates	1 mm	0.6 (NS) 95% CI (–1.6 to 2.8)	0.02 (*P* < 0.001) 95% CI (0.01–0.03)	–2.0 (*P* = 0.03) 95% CI (–3.8 to –0.2)
SRF	Adjusted for age and sex	1 mm	–2.5 (NS) 95% CI (–6.0 to 0.9)	0.0 (NS) 95% CI (–0.01 to 0.00)	–4.3 (*P* < 0.001) 95% CI (–6.4 to –2.1)
	Adjusted for all covariates	1 mm	0.2 (NS) 95% CI (–1.6 to 2.8)	0.01 (NS) 95% CI (0.01–0.00)	–3.3 (*P* < 0.001) 95% CI (–5.0 to –1.5)
PED	Adjusted for age and sex	1 mm	–1.4 (NS) 95% CI (–2.9 to 0.2)	0.01 (*P* < 0.001) 95% CI (0.01–0.01)	–1.9 (*P* < 0.001) 95% CI (–2.9 to –0.9)
	Adjusted for all covariates	1 mm	–0.3 (NS) 95% CI (–1.3 to 0.6)	0.01 (*P* < 0.001) 95% CI (0.01–0.01)	–1.5 (*P* < 0.001) 95% CI (–2.3 to –0.6)
IRF	Adjusted for age and sex	6 mm	–2.1 (*P* < 0.001) 95% CI (–3.2 to –1.1)	0.09 (*P* < 0.001) 95% CI (0.05–0.13)	–1.0 (*P* < 0.001) 95% CI (–1.5 to –0.6)
	Adjusted for all covariates	6 mm	0.5 (NS) 95% CI (–0.2 to 1.2)	0.07 (*P* = 0.004) 95% CI (0.01–0.01)	–0.4 (NS) 95% CI (–0.8 to 0.1)
SRF	Adjusted for age and sex	6 mm	–0.4 (*P* = 0.01) 95% CI (–0.7 to –0.1)	0.0. (NS) 95% CI (–0.01 to 0.02)	–0.6 (*P* < 0.001) 95% CI (–0.7 to –0.4)
	Adjusted for all covariates	6 mm	0.3 (*P* = 0.007) 95% CI (0.1–0.5)	0.01 (NS) 95% CI (–0.02 to 0.01)	–0.4 (*P* < 0.001) 95% CI (–0.5 to –0.3)
PED	Adjusted for age and sex	6 mm	0.0 (NS) 95% CI (–0.1 to 0.1)	0.02 (*P* < 0.001) 95% CI (0.02–0.03)	–0.1 (*P* < 0.001) 95% CI (–0.2 to 0.0)
	Adjusted for all covariates	6 mm	0.0 (NS) 95% CI (–0.1 to 0.1)	0.02 (*P* < 0.001) 95% CI (0.02–0.03)	–0.1 (*P* < 0.001) 95% CI (–0.2 to –0.1)

BCVA = best-corrected visual acuity; CI = confidence interval; EZ= ellipsoid zone; IRF= intraretinal fluid; NS = not significant; PED = pigment epithelial detachment; SRF= subretinal fluid.

To account for the multicenter design, linear mixed-effects models including a random intercept for study center were fitted. These models demonstrated modest site-level variability (standard deviation ≈ 3.4 letters for BCVA) but confirmed the same pattern of associations: IRF remained independently associated with BCVA at month 1, whereas IRF and PED remained associated with EZ thinning and loss.

Interreader agreement for EZ loss quantification showed reliability with an ICC (2,1) of 0.88 (95% CI, 0.80–0.91), improving to 0.93 (95% CI, 0.89–0.95) when averaged across readers. For layer thickness measurements, agreement was also excellent at the single-reader level, ICC (2,1) = 0.92; 95% CI, 0.88–0.94, and for average measurements, ICC (2,k) = 0.96; 95% CI, 0.92–0.97. Confidence intervals were narrow for all ICC estimates, indicating measurement stability. A Bland-Altman plot for EZ thickness measurements was added as Supplementary Material S1 (available at www.ophthalmologyscience.org).

## Discussion

In our study, we performed a systematic correlation of retinal fluid volumes by compartment and distribution with parameters of retinal function and connected the structure–function correlation with the underlying condition of the EZ layer. The clinically relevant biomarkers in nAMD, such as IRF, SRF, PED volumes, as well as EZ thickness and loss, were accurately quantified to provide robust correlations. A large population from a clinical setting was used with evidence of active nAMD.

The correlation between fluid amounts and retinal function is discussed widely in the community with no clear conclusions. Providing a comprehensive analysis may bridge the gap between anatomic alteration and functional deficits. A significant influence of retinal fluid volumes at baseline on both visual and anatomic outcomes was found, consistent with the related “functional” biomarker evidence. The results demonstrate that higher fluid volumes at baseline, particularly in the intraretinal compartment, are correlated with greater photoreceptor damage and, consequently, early visual loss, underscoring the importance of compartment-specific and personalized fluid management in optimizing treatment. Innovative AI-based tools to assess measurable biomarkers such as EZ thickness and EZ loss have gained more importance, particularly as early indicators of MA. However, comprehensively combining fluid- as well as EZ-related biomarker analysis in a quantitative manner has not been performed in relevant nAMD populations.

Our study population was shown to be representative for a clinical setting. The observation of differential fluid compartment volumes between MNV subtypes in this study is consistent with previous work that emphasizes that each subtype (1, 2, and 3) has distinct fluid patterns and prognostic implications.^31^^,^^32^ Furthermore, Sharma et al^33^ suggested even more distinct fluid patterns associated with MNV type 3 and potential implications for treatment response. In our study, MNV type 3 was the second most prevalent type and was associated with a lower amount of SRF. Interestingly, SRF in general has been linked to a lower incidence of MA and subretinal fibrosis compared with IRF.^34^ This condition does not seem to apply to type 3, suggesting another pathophysiology in this specific entity as previously mentioned in the literature.^35^

We selected baseline and month 1 conditions to isolate the direct effect of fluid on function because the change in fluid volumes is most substantial after the first treatment, the EZ parameters can reliably be quantified, and any subsequent effect by late complications such as fibrosis or macular atrophy can be better identified. The association between baseline fluid volumes and BCVA at month 1 in this study aligns with previous research and underscores the prognostic significance of fluid characteristics in nAMD. Overall, high baseline fluid volumes were shown to negatively impact functional outcomes independently of the drug and regimen, with more fluid for each compartment leading to more visual loss.^36^ Most importantly, we found that IRF in both the central 1 and 6 mm at baseline was associated with a decreased BCVA after 1 month. This finding corroborates the concept that IRF contributes more to visual impairment than other fluid compartments.^37^ Studies using other real-world cohorts also reported this overriding association.^21^^,^^22^^,^^38^ However, the global correlation between retinal fluid amount and BCVA still misses the evidence of underlying anatomic degradation of photoreceptors, which is the anatomic correlate for visual impairment.^39^^,^^40^ This study demonstrated that IRF had the most pronounced effect on EZ thinning and EZ loss, indicating that IRF is directly leading to alteration of the structure and function of photoreceptors. Furthermore, it is important to underscore that not only fluid volume and fluid location play a role, but fluid dynamics also impact patients' outcomes via this direct path.^17^

The present study observed that SRF was not statistically significantly associated with BCVA loss or EZ loss in the central 1- and 6 mm area. However, SRF was associated with a slight increase in EZ thickness in the central 6 mm region, an association that was not present in the central 1 mm. Clinically, it is important to note that SRF volumes in the central 1 mm of the macula are usually low,^41^ as was also seen in our cohort. Moreover, it is important to highlight the potential effect of an extension of the interdigitation zone in the presence of SRF, which could lead to an increase in the physiological EZ thickness measurements as the segmentation spans from the inner boundary of the EZ to the outer boundary of the interdigitation zone. There is still an open question in the literature whether SRF is less harmful. The Comparison of AMD Treatment Trials study showed that persistent SRF did not affect visual outcomes significantly through 1 year of follow-up.^38^

Pigment epithelial detachment volumes showed no significant impact on BCVA in this analysis. However, high PED volumes were significantly correlated with EZ thinning and loss. Interestingly, our findings also showed that increased PED volumes were associated with decreased baseline retinal sensitivity and that retinal sensitivity was independently associated with EZ loss at month 1, underscoring the role of early structural–function correlation in nAMD. Nevertheless, a correlation between higher PED volumes after initial treatment and worse visual outcomes in a long-term follow-up was demonstrated in previous studies from our group.^21^

Fibrovascular sub-RPE pathology is likely linked to overlying retinal alteration, with photoreceptors suffering from degenerative processes. It has been shown that in clinical trials and real-world studies, up to 98% of patients with nAMD demonstrate MA over time, which was defined as loss of RPE, outer retina, and choriocapillaris.^42^

In this study, eyes with central 1-mm macular atrophy, subfoveal fibrosis, or both at baseline were excluded to avoid inclusion of advanced disease stages that could confound assessment of treatment response. Nevertheless, subtle outer retinal alterations or early fibrotic changes may already be present at baseline, especially in pretreated patients, and remain partially masked by fluid volume or retinal hemorrhage. Therefore, the structural changes observed during the early follow-up period may reflect preexisting outer retinal damage.

The robust design of this study as a prospective clinical trial, combined with a large sample size, enhances the statistical power of the findings, providing a higher level of confidence in the reliability and validity of the results. All analyses were conducted independently by the academic investigators. The industrial partner contribution was limited to providing access to the AI algorithm and technical support for image export. The present analysis has 2 main limitations: the short follow-up period between baseline and month 1 and the relatively low *R*^2^ values observed in the multivariable models. The short-term analysis was intentionally designed to capture an association of the anatomic and functional response to treatment. Long-term follow-up analyses are ongoing and will include the assessment of long-term visual and anatomic outcomes, as well as treatment frequency. The availability of a large and standardized clinical setting data set provides a unique opportunity to investigate both early predictors and durable outcomes in nAMD.

Moreover, lower *R*^2^ values are not uncommon in visual acuity studies, reflecting the multifactorial nature of visual outcomes in retinal diseases.

In conclusion, this study demonstrates a significant association of fluid type and volume on photoreceptor function by quantifying fluid and EZ-related parameters. Clinically, it contributes to the growing body of evidence supporting the utility of AI-based fluid monitoring in nAMD management. By corroborating and extending findings from previous literature, our results highlight the importance of fluid characterization in treatment response and visual outcomes, thereby facilitating personalized treatment approaches for patients with nAMD.
