# Supplementary material for: Early Structure–Function Correlates in Neovascular Age-Related Macular Degeneration Using Artificial Intelligence–Derived OCT Biomarkers
Source: Ophthalmol Sci. 2026 Jun 10;6(9):101281. doi: 10.1016/j.xops.2026.101281 (PMC13487276; doi:10.1016/j.xops.2026.101281)
Supplement: Supplementary file 1 [file mmc1.pdf]

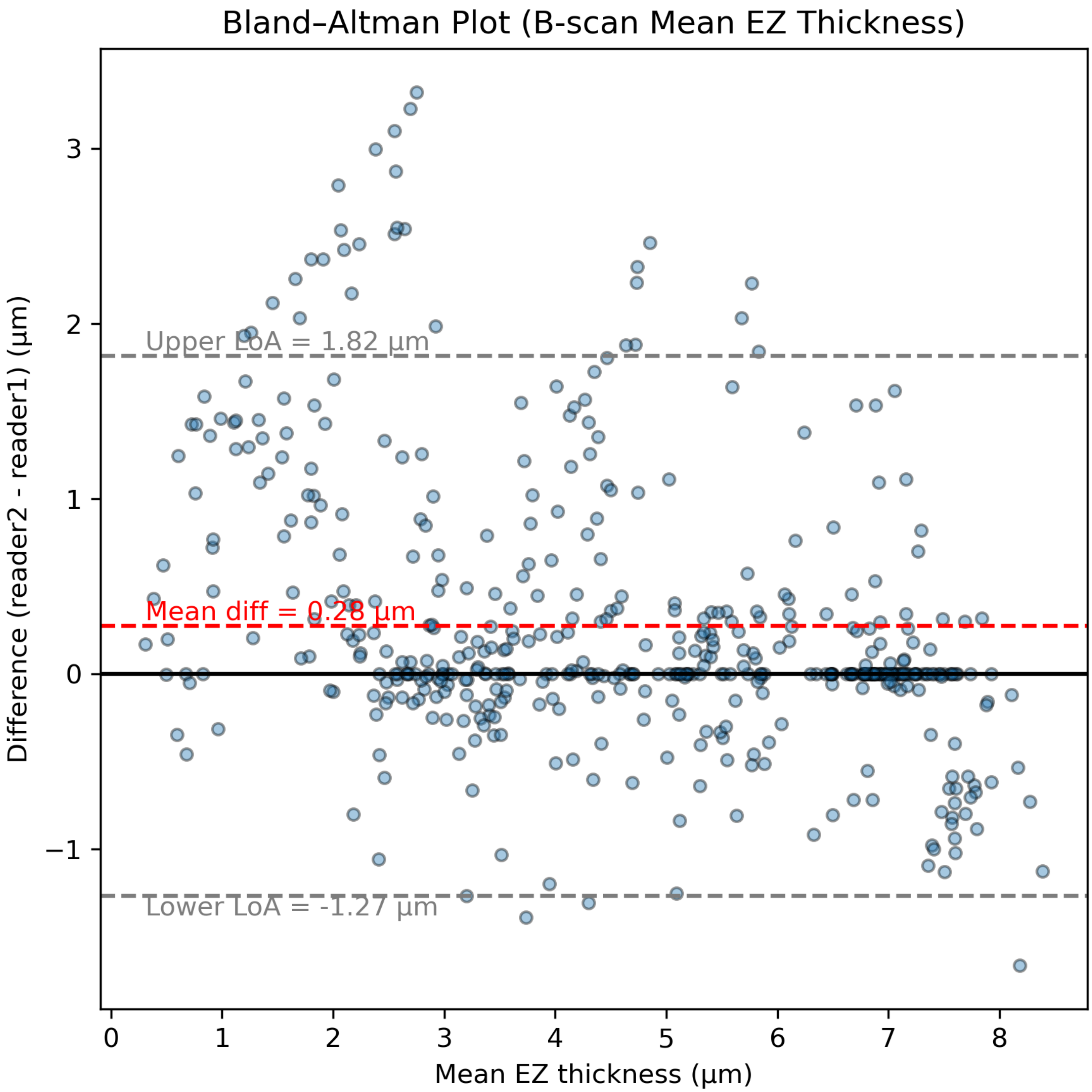

Bland–Altman Plot (B-scan Mean EZ Thickness)
Upper LoA = 1.82 µm
Mean diff = 0.28 µm
Lower LoA = -1.27 µm
Difference (reader2 - reader1) (µm)
Mean EZ thickness (µm)
3
2
1
0
−1
0
1
2
3
4
5
6
7
8
