# Supplementary material for: Early Structure–Function Correlates in Neovascular Age-Related Macular Degeneration Using Artificial Intelligence–Derived OCT Biomarkers
Source: Ophthalmol Sci. 2026 Jun 10;6(9):101281. doi: 10.1016/j.xops.2026.101281 (PMC13487276; doi:10.1016/j.xops.2026.101281)
Supplement: Supplementary file 2 [file mmc2.pdf]

Paired spaghetti plot: BCVA baseline to month 1, stratified by fluid compartment volumes, shows individual patient trajectories in BCVA

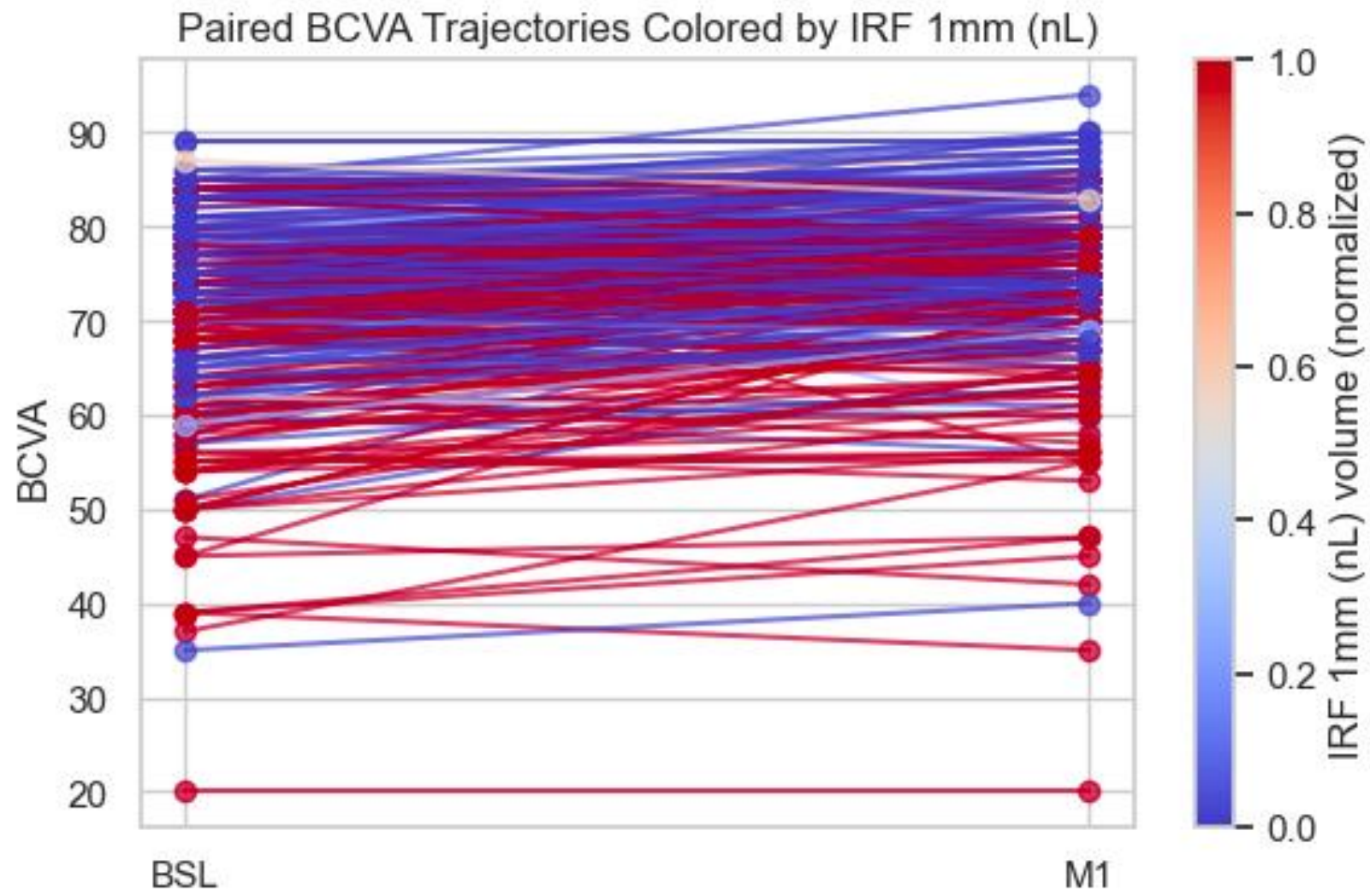


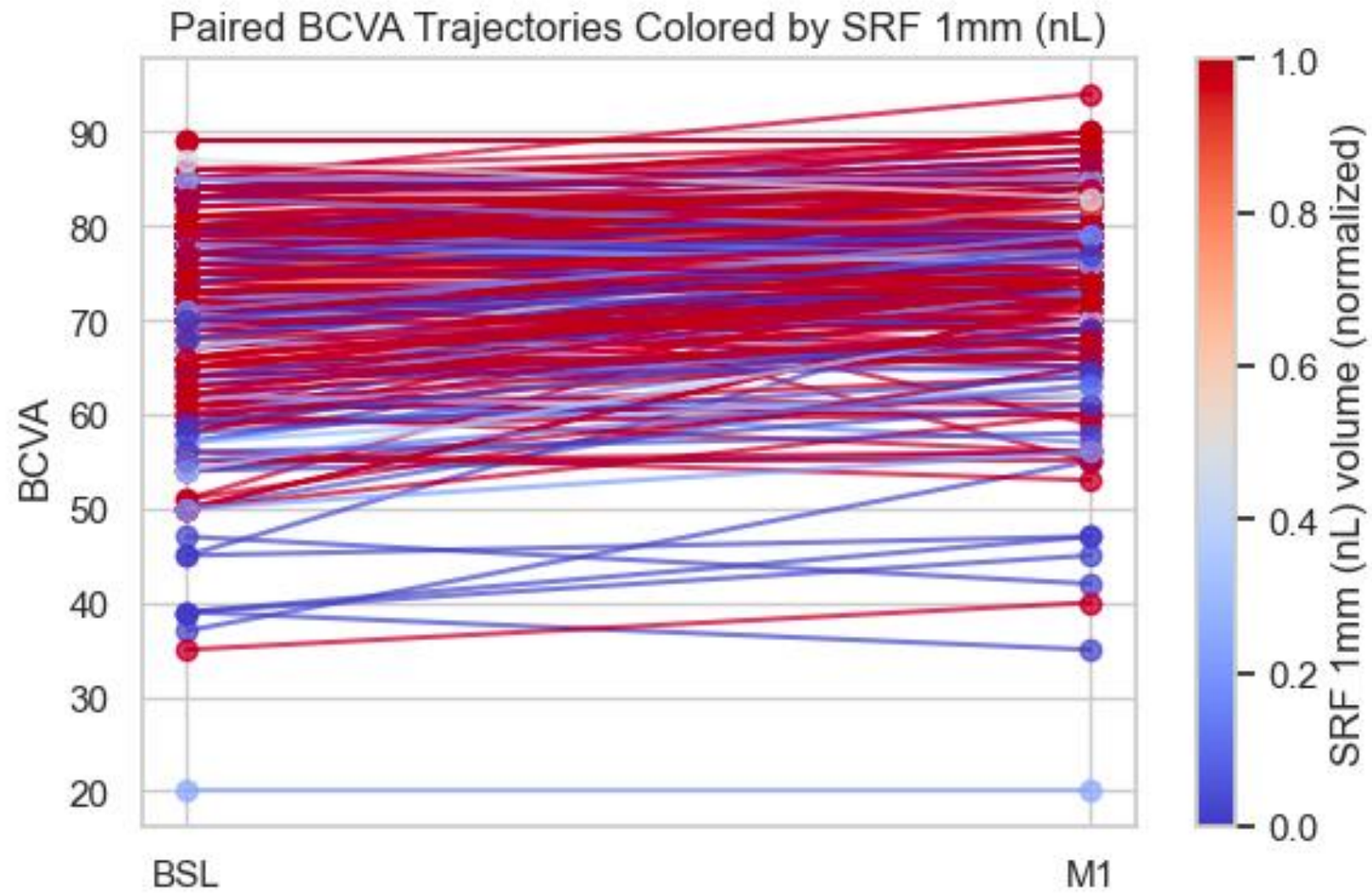


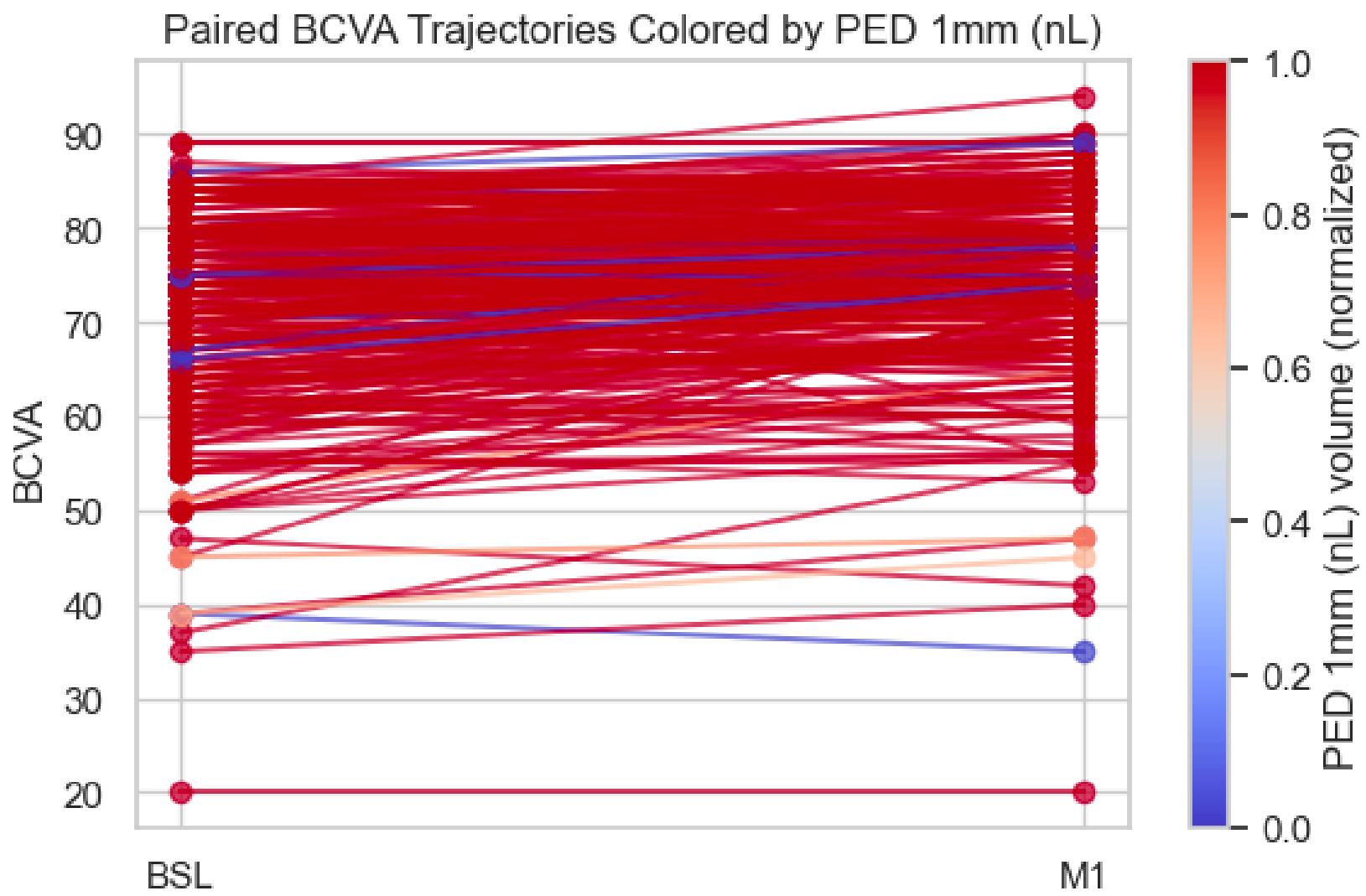

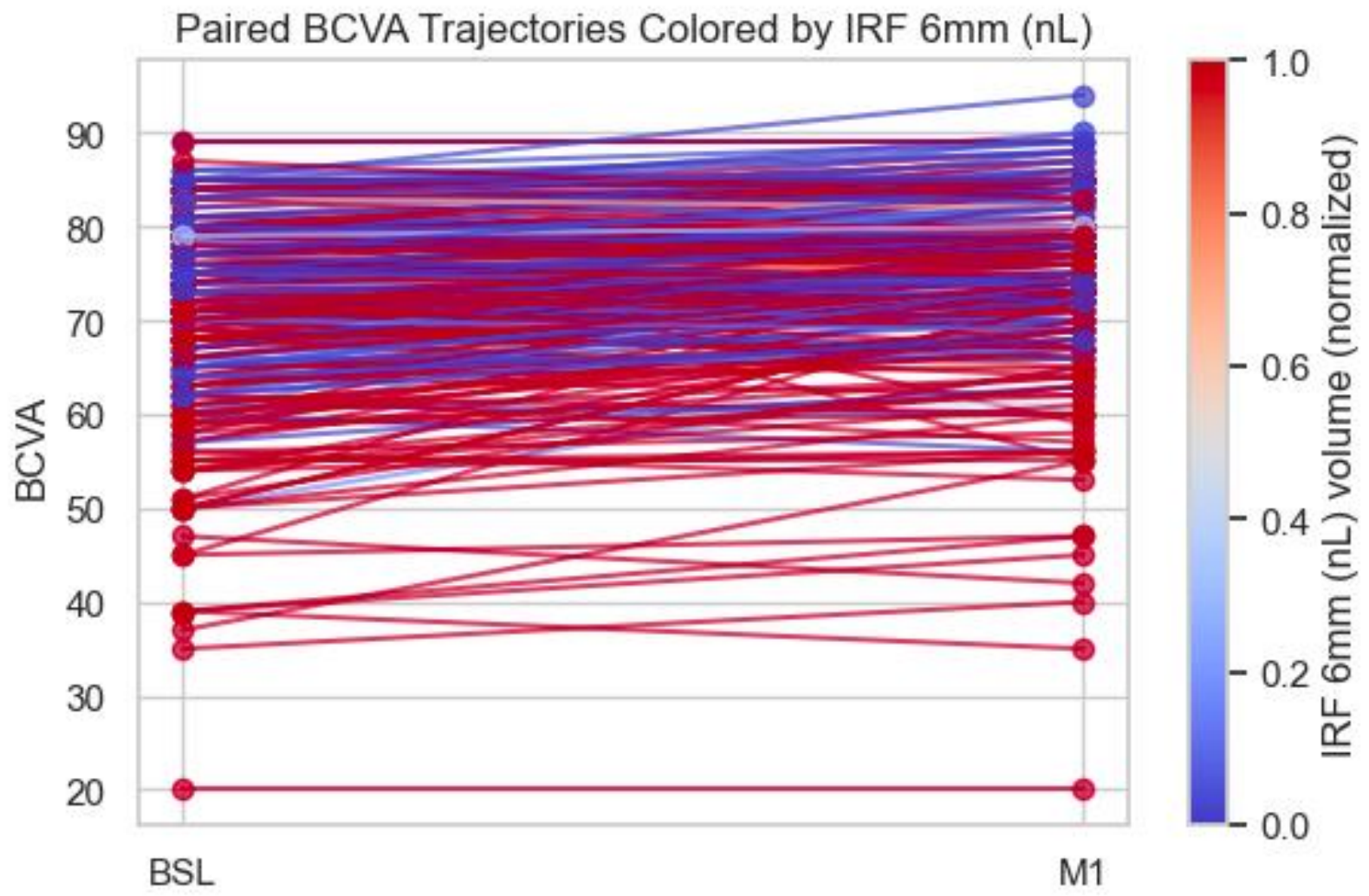
Paired BCVA Trajectories Colored by IRF 6mm (nL)
BCVA
20
30
40
50
60
70
80
90
BSL
M1
IRF 6mm (nL) volume (normalized)
0.0
0.2
0.4
0.6
0.8
1.0

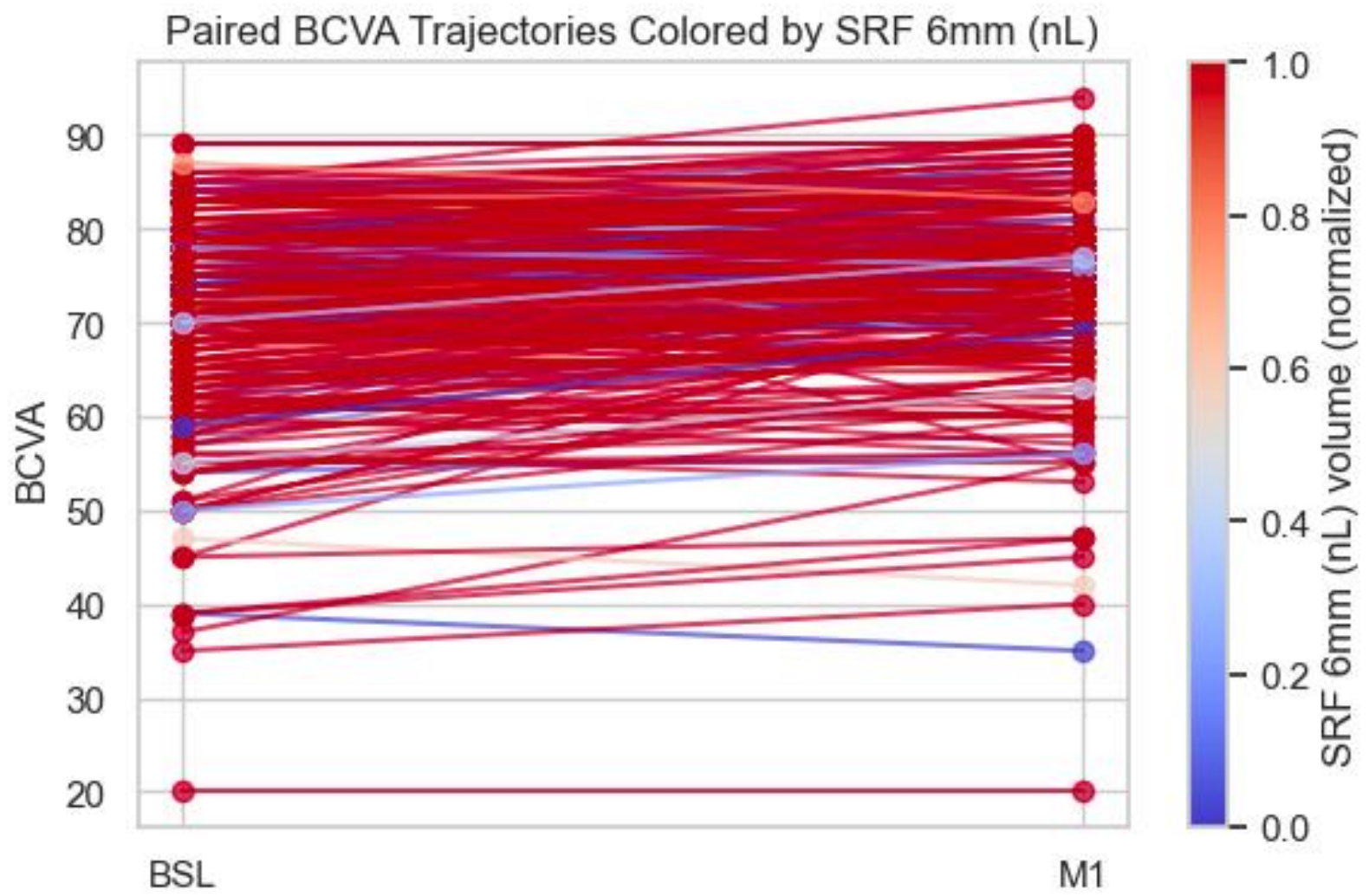
Paired BCVA Trajectories Colored by SRF 6mm (nL)
BCVA
20
30
40
50
60
70
80
90
BSL
M1
SRF 6mm (nL) volume (normalized)
0.0
0.2
0.4
0.6
0.8
1.0

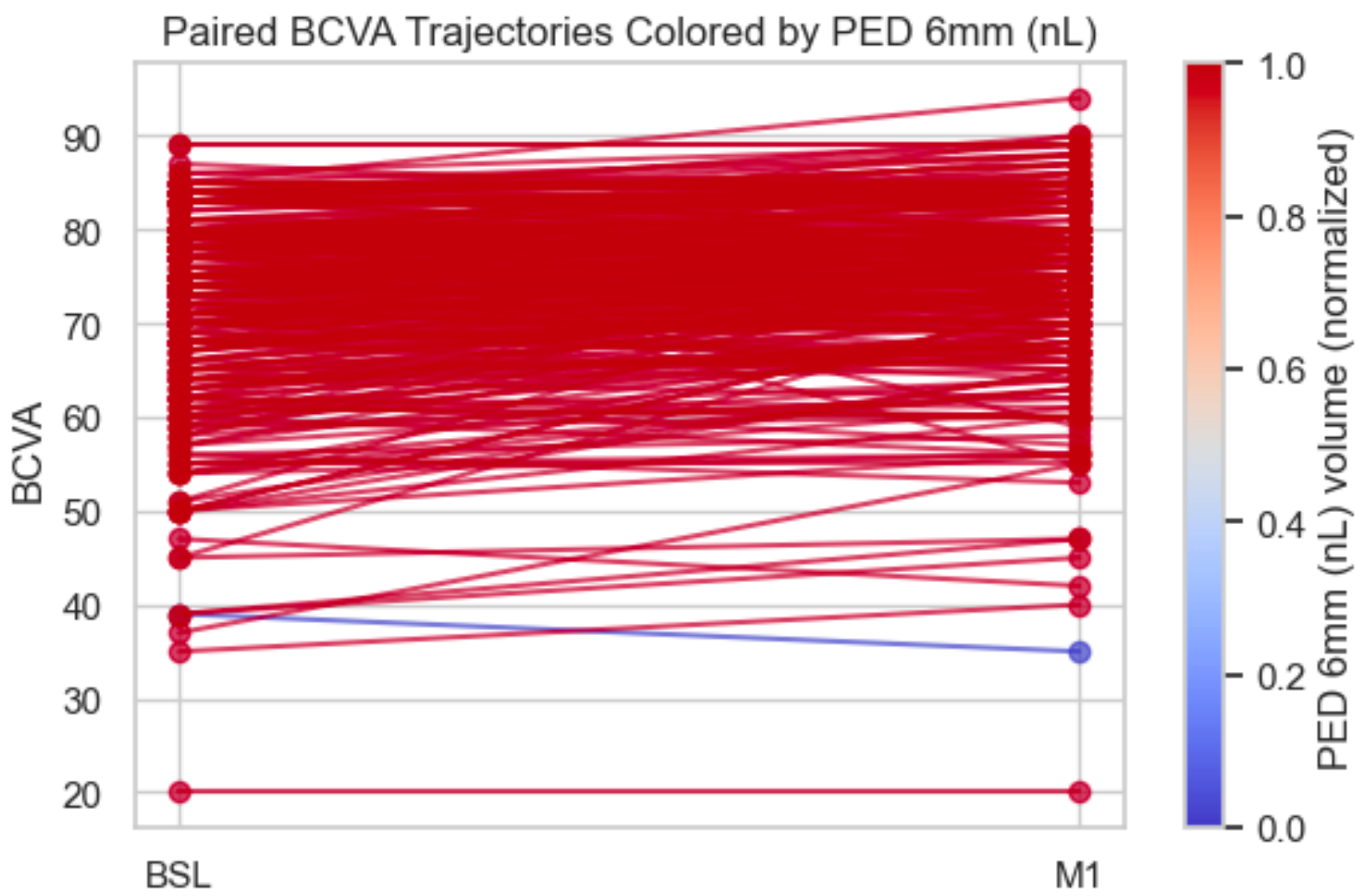
Paired BCVA Trajectories Colored by PED 6mm (nL)
BCVA
20
30
40
50
60
70
80
90
BSL
M1
PED 6mm (nL) volume (normalized)
0.0
0.2
0.4
0.6
0.8
1.0

Raincloud plots for EZ thickness and loss per quartiles.

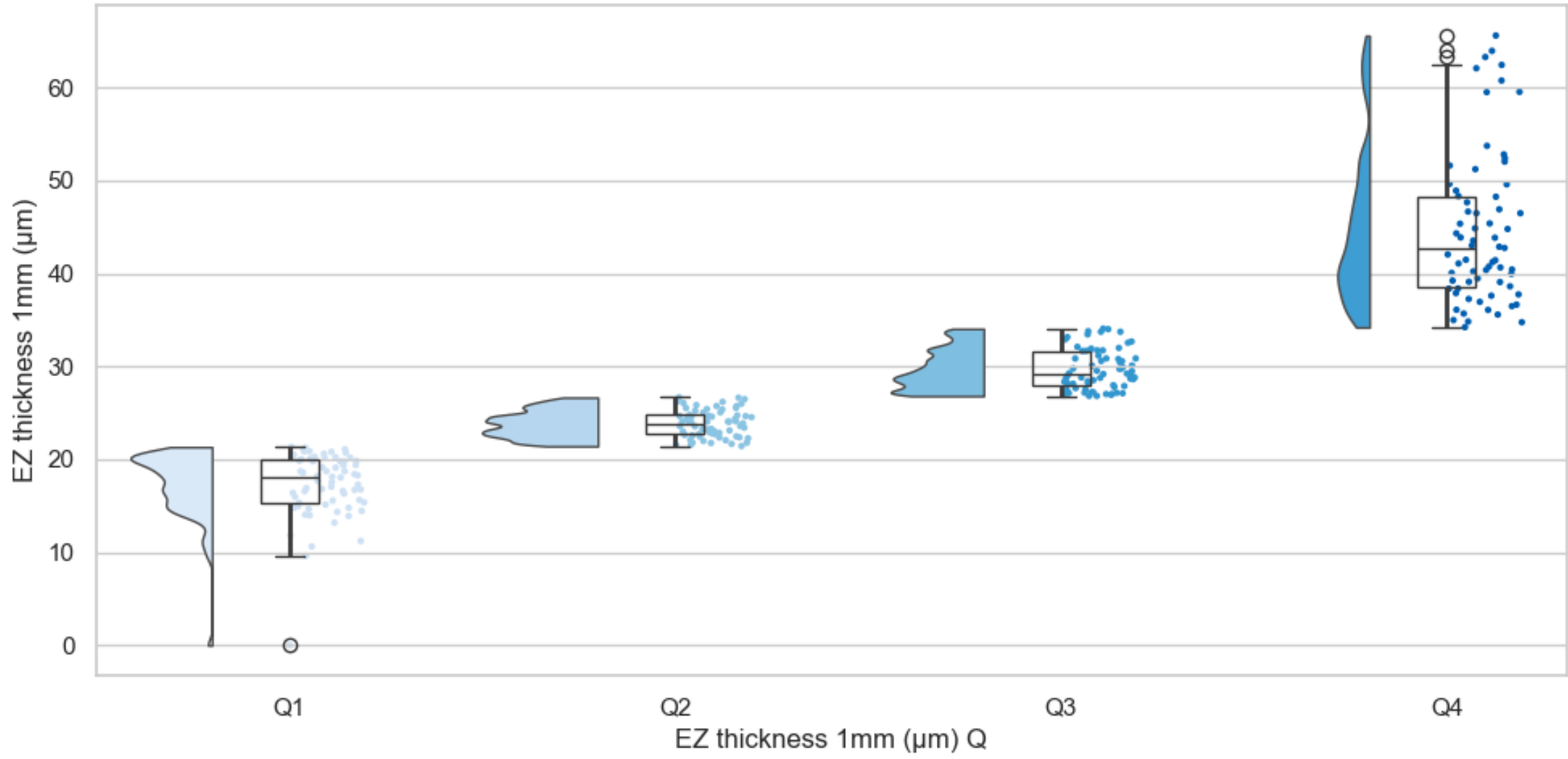


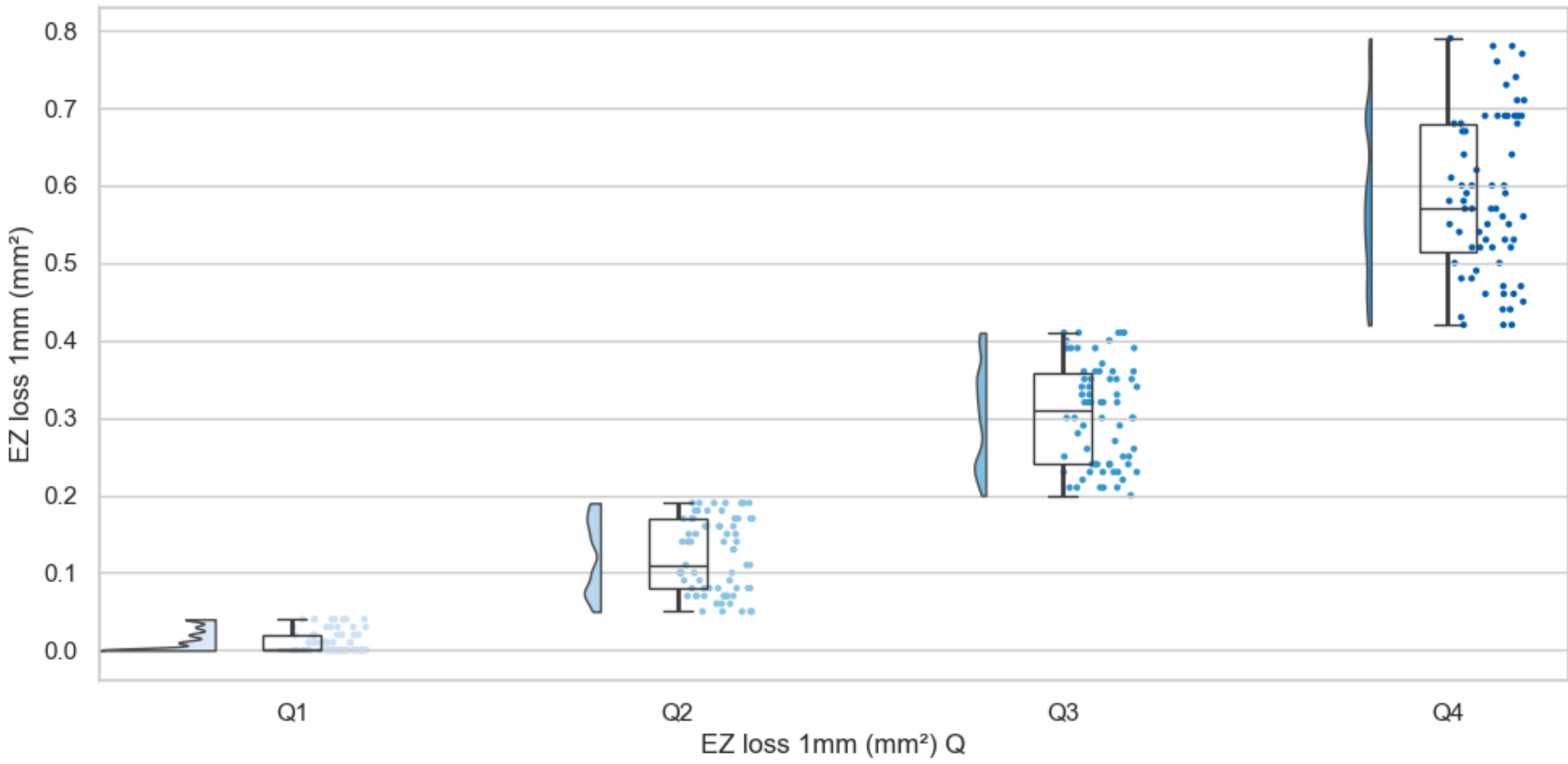

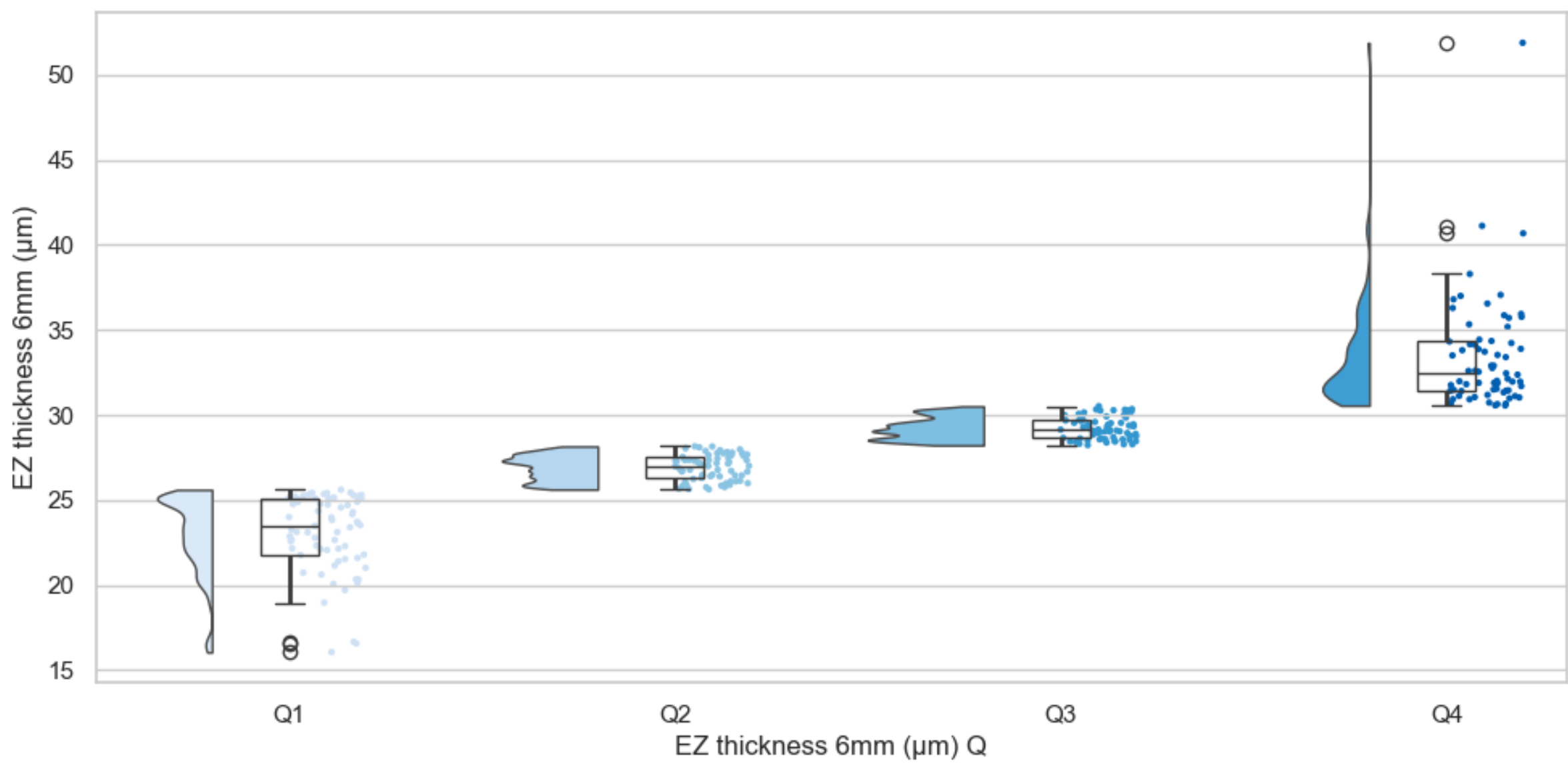

EZ thickness 6mm (μm)
50
45
40
35
30
25
20
15
Q1
Q2
Q3
Q4
EZ thickness 6mm (μm) Q


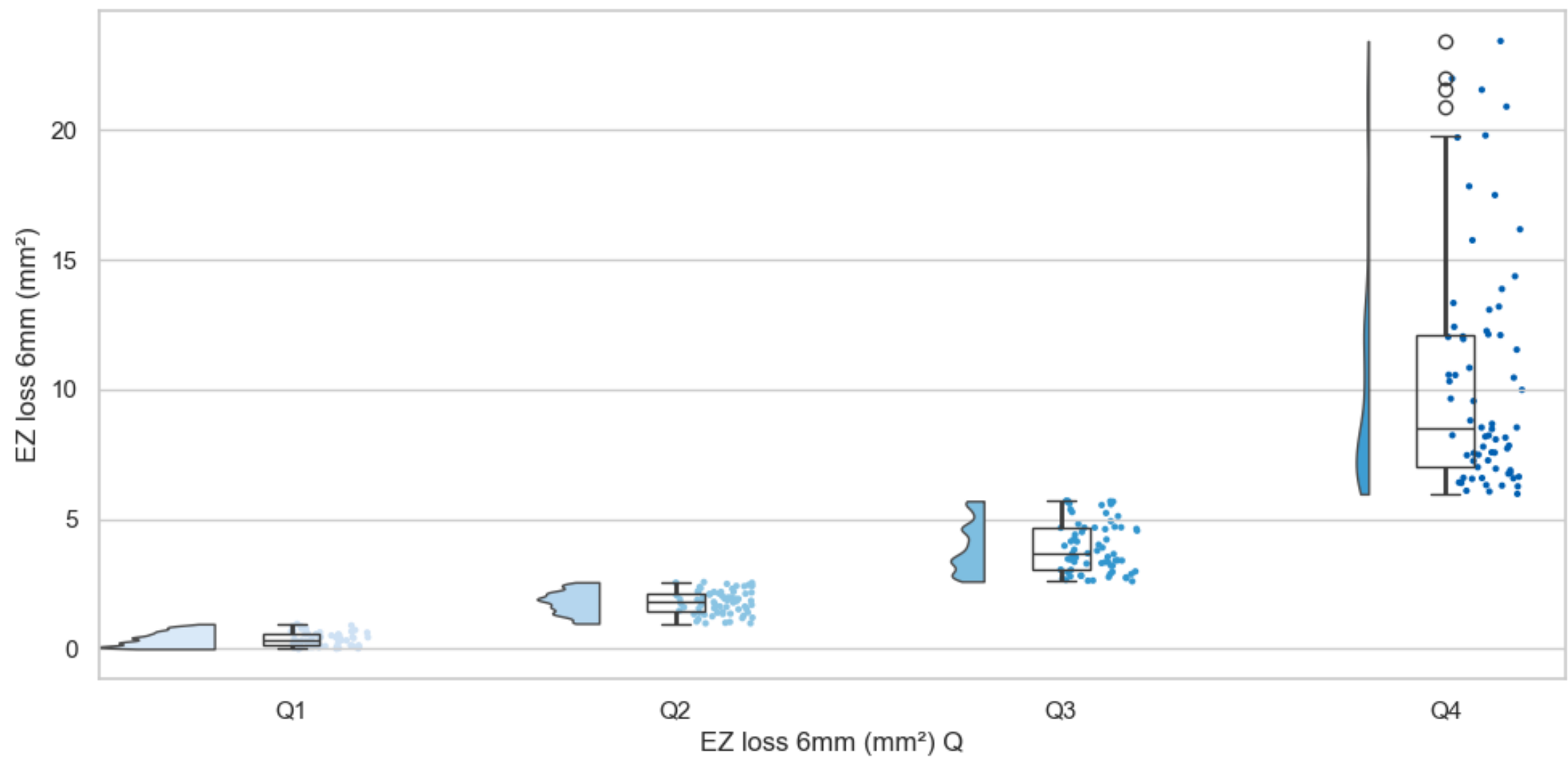

EZ loss 6mm (mm²)
20
15
10
5
0
Q1
Q2
Q3
Q4
EZ loss 6mm (mm²) Q
